# Current concepts regarding Graves’ orbitopathy

**DOI:** 10.1111/joim.13524

**Published:** 2022-06-01

**Authors:** Luigi Bartalena, Maria Laura Tanda

**Affiliations:** ^1^ Department of Medicine and Surgery University of Insubria Varese Italy

**Keywords:** glucocorticoids, Graves’ orbitopathy, iscalimab, rituximab, teprotumumab, thyrotropin receptor, tocilizumab, TSH receptor

## Abstract

Graves’ orbitopathy (GO) is an orbital autoimmune disorder and the main extrathyroidal manifestation of Graves’ disease, the most common cause of hyperthyroidism. GO affects about 30% of Graves’ patients, although fewer than 10% have severe forms requiring immunosuppressive treatments. Management of GO requires a multidisciplinary approach. Medical therapies for active moderate‐to‐severe forms of GO (traditionally, high‐dose glucocorticoids) often provide unsatisfactory results, and subsequently surgeries are often needed to cure residual manifestations. The aim of this review is to provide an updated overview of current concepts regarding the epidemiology, pathogenesis, assessment, and treatment of GO, and to present emerging targeted therapies and therapeutic perspectives. Original articles, clinical trials, systematic reviews, and meta‐analyses from 1980 to 2021 were searched using the following terms: Graves’ disease, Graves’ orbitopathy, thyroid eye disease, glucocorticoids, orbital radiotherapy, rituximab, cyclosporine, azathioprine, teprotumumab, TSH‐receptor antibody, smoking, hyperthyroidism, hypothyroidism, thyroidectomy, radioactive iodine, and antithyroid drugs. Recent studies suggest a secular trend toward a milder phenotype of GO. Standardized assessment at a thyroid eye clinic allows for a better general management plan. Treatment of active moderate‐to‐severe forms of GO still relies in most cases on high‐dose systemic—mainly intravenous—glucocorticoids as monotherapy or in combination with other therapies—such as mycophenolate, cyclosporine, azathioprine, or orbital radiotherapy—but novel biological agents—including teprotumumab, rituximab, and tocilizumab—have achieved encouraging results.

## Graphical abstract

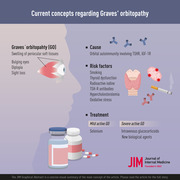

**Abbreviations**
ACE: angiotensin‐converting enzyme
ATD: antithyroid drug
CAS: clinical activity score
DON: dysthyroid optic neuropathy
ETA: European Thyroid Association
EUGOGO: European Group on Graves’ Orbitopathy
GCs: glucocorticoids
GO: Graves’ orbitopathy
IGF‐1: insulin‐like growth factor‐1
IGF‐1R‐Ab: IGF‐1 receptor antibodies
IL‐6: interleukin‐6
ivGCs: intravenous glucocorticoids
OR: orbital radiotherapy
RAI: radioactive iodine
RCT: randomized clinical trial
TSHR: TSH receptor
TSHR‐Ab: TSHR antibodies

## Introduction

Graves’ orbitopathy (GO) is an orbital autoimmune disease constituting the most frequent extrathyroidal expression of Graves’ disease [1]. Full‐blown disease is associated with disfiguring features (exophthalmos, stare), inflammatory signs and symptoms, ocular dysfunction (diplopia), and, rarely, visual loss due to compressive dysthyroid optic neuropathy (DON) [1] (Table 1). GO heavily affects quality of life and bears relevant public health consequences, including sick leave, work disability, and costs of therapies [2, 3, 4].

**Table 1:** Main symptoms and signs of Graves’ orbitopathy

**Symptoms**	
Lacrimation	
Grittiness (sandy sensation)	
Photophobia	
Ocular pain (spontaneous or with eye movements)	
Diplopia (intermittent, when tired or awakening; inconstant, at extremes of gaze; constant, in all positions of gaze)	
Decreased color sensitivity	
Visual loss	
**Signs**	
Lid retraction (stare)	
Periocular soft tissue swelling and redness	
Conjunctival hyperemia and edema (chemosis)	
Exophthalmos (bulging eyes)	
Lagophthalmos (incomplete eye closure at night)	
Decreased ocular motility/strabismus	
Decreased visual acuity (due to dysthyroid optic neuropathy)	

Relevant progress has been made in the last 20 years in the understanding of GO epidemiology and pathogenesis, the identification of risk factors, the standardized assessment of patients, and general treatment plan. Novel biological agents have been introduced for GO targeted treatment, with promising results. These recent achievements are presented in this review.

### Epidemiology

The estimated incidence of GO—based on registry studies [5, 6]—is 3.3–8.0/100,000/year in women and 0.9–1.6/100,000/year in men [7], lower than previously reported [8]. The estimated prevalence of GO in the general population is around 9/10,000 population [9]. Thus, although uncommon, GO does not fulfill—with the exception of the variant euthyroid GO—the major criterion of prevalence of less than 5/10,000 population to be designated as a rare disease [9].

**Fig. 1:**
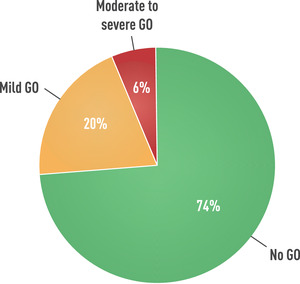
Prevalence of Graves’ orbitopathy in patients with newly diagnosed, recent‐onset Graves’ hyperthyroidism. Derived from Tanda et al. [12].

The prevalence of GO among Graves’ patients is around 30% [10, 11]. In a single‐center study of more than 300 consecutive patients with recent onset Graves’ hyperthyroidism, 74% had no signs/symptoms of GO, 20% had mild GO, and only 6% had moderate‐to‐severe or, rarely, sight‐threatening GO [12, 13] (Fig. 1). Interestingly, consecutive patients referred to tertiary centers of the European Group on Graves’ Orbitopathy (EUGOGO) in 2012 had less severe and active GO than in 2000 [14]. Lower prevalence and severity of GO may reflect a secular trend toward a milder phenotype of Graves’ disease [11, 13]. Earlier diagnosis and treatment of both hyperthyroidism and GO, and other factors—including decreased smoking and micronutrient supplementation—likely play a fundamental role [11].

### Pathogenesis

GO is mostly associated with Graves’ hyperthyroidism, although in 7%–8% of cases it may occur with euthyroid/hypothyroid chronic autoimmune thyroiditis [1]. As Graves’ disease is due to loss of tolerance to TSH receptor (TSHR), GO may be triggered by autoimmune reactions against TSHR [15], as supported by experimental and clinical evidence [16]. After initial demonstration of TSHR expression in orbital tissue [17, 18], immunohistochemical studies have shown TSHR overexpression in GO orbital tissues [19, 20, 21]. TSHR activation enhances the differentiation of orbital preadipocytes into adipocytes, favoring the expansion of orbital adipose tissue [22, 23]. The key role of TSHR is supported by recent animal models obtained in rodents [24, 25, 26, 27]. From a clinical standpoint, circulating TSHR antibodies (TSHR‐Ab) correlate with GO activity [28, 29, 30]. In a prospective study of consecutive GO patients, novel cell‐based TSHR‐Ab bioassays measuring stimulatory TSHR‐Ab showed higher sensitivity and specificity than commonly used immunoassays in differentiating clinically active from inactive, and mild from moderate‐to‐severe, GO [31]. Finally, TSHR‐Ab is an independent risk factor for GO and may predict GO severity and outcome [32]. Therefore, solid evidence links TSHR and stimulatory TSHR‐Ab to GO development. TSHR‐Ab can be considered a biomarker of GO [33, 34].

Insulin‐like growth factor‐1 (IGF‐1) receptor (IGF‐1R) is another important player [35, 36, 37]. B cells [38] and T cells [39] from Graves’ patients overexpress IGF‐1R; Graves’ immunoglobulins stimulate hyaluronan synthesis by orbital fibroblasts through IGF‐1R [40] and induce orbital fibroblast proliferation and cytokine secretion [41]. TSHR and IGF‐1R colocalize on orbital fibroblasts and thyrocytes [42]. IGF‐1R activation on orbital fibroblasts may follow the binding of stimulatory IGF‐1R antibodies (IGF‐1R‐Ab) to IGF‐1R. Alternatively, IGF‐1R activation may result from synergistic cross‐talk between TSHR and IGF‐1R after the binding of stimulatory TSHR‐Ab to TSHR, causing activation of IGF‐1R‐dependent intracellular signaling pathways [43]. Consistent with this hypothesis, teprotumumab—an IGF‐1R‐blocking monoclonal antibody that does not bind to TSHR—inhibited IGF‐1R‐dependent production of hyaluronan induced by a monoclonal stimulatory TSHR‐Ab (M22), but not IGF‐1R‐independent M22 stimulation [44]. There is no animal model of GO employing IGF‐1R for immunization. From a clinical standpoint, after initial demonstration of Graves’ immunoglobulins displacing IGF‐1 bound to GO orbital fibroblasts [45], subsequent studies have provided conflicting results, showing that a subset of patients with GO may have circulating IGF‐1R‐Ab with either inhibitory [46] or stimulatory IGF‐1R functional properties [47]. More recently, a pilot study using a novel IGF‐1R‐Ab assay showed that Graves’ patients without GO had higher serum IGF‐1R‐Ab concentrations than those with GO [48], suggesting a putative “protective” role of IGF‐1R‐Ab for GO. For the time being, the role of IGF‐1R‐Ab in GO pathogenesis is speculative.

Fibroblasts are the primary victims of the autoimmune attack [49]. Orbital fibroblasts also include a subset of CD34+ bone‐marrow‐derived fibrocytes expressing thyroid antigens, including TSHR and thyroglobulin [50]. Serum thyroglobulin levels are higher in Graves’ patients with GO than in those without [51], but whether thyroglobulin is another thyroid antigen involved in GO pathogenesis is not settled. B cells, dendritic cells, and T cells—activated after presentation of thyroid autoantigen(s)—infiltrate the orbit and produce proinflammatory cytokines, growth factors, and chemoattractants [20, 49]. In immunohistochemical studies, both T cells (CD3+ and CD4+) and B cells (CD20+) are often organized into distinct foci and associated with GO activity [52, 53]. B cells work as antigen‐producing cells and autoantibody‐producing cells [49]. Interaction of T cells with orbital fibroblasts, as well as the binding of TSHR‐Ab—and possibly IGF‐1R‐Ab—produced by B cells, activates the TSHR‐IGF‐1R complex. As a consequence, orbital fibroblasts proliferate—further releasing inflammatory molecules—but also differentiate into adipocytes, thereby eventually causing an increase in the orbital fibro‐adipose tissue, while extraocular muscles are infiltrated by inflammatory cells [49]. Orbital thyroid tissue from GO patients under‐ and overexpress numerous genes [54, 55, 56]. Genetic profiling of GO and control fibroblasts confirmed that some of the underexpressed genes encode proteins involved in the downregulation of cell growth, apoptosis, and antioxidant activity, while some of the overexpressed genes encode proteins enhancing cell growth and inhibiting proptosis [57]. In addition, after autoimmune reactions have been triggered, orbital fibroblasts produce glycosaminoglycans—mainly hyaluronan—which are hydrophilic and attract water, causing edema in orbital tissue and extraocular muscle. The increase in orbital volume and edema in a limited space such as the orbit mechanically explain manifestations of GO including exophthalmos, periocular soft tissue swelling, extraocular muscle enlargement/dysfunction, and optic nerve compression [58].

### Risk factors and prevention

**Fig. 2:**
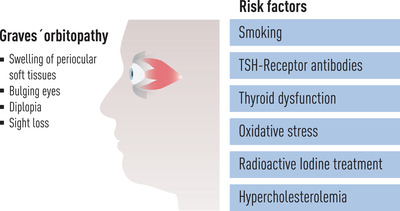
Modifiable risk factors for de novo occurrence or progression of Graves’ orbitopathy.

GO results from a complex interplay between endogenous (nonmodifiable) and exogenous (modifiable) risk factors [7] (Fig. 2).

GO is rare in childhood [59]. Moderate‐to‐severe GO is less frequent in patients younger than 40 years, with the mean age of patients with severe forms of GO being 50–56 years [6, 12]. Although more frequent in women, gender difference attenuates in severe GO. In a study of more than 2000 Graves’ patients, clinically relevant GO was found in about half of women and men, but patients with moderate‐to‐severe GO were more commonly men (30% vs. 21%) and older [60]. Thus, men tend to have more severe GO at an older age [7, 61, 62].

The role of genetic factors conferring predisposition to Graves’ disease has long been proposed, based on twin and family studies, and family clustering of thyroid autoimmune disorders [63, 64, 65]. Several candidate genes have been indicated, but the differentiation of the genetic profile of Graves’ patients with or without GO is uncertain [66, 67]. Additionally, in twin studies the concordance rate is only about 30%, suggesting a low penetrance of involved genes [63, 68]. Epigenetic factors—including abnormal DNA methylation, histone modification, and noncoding RNAs—may contribute to GO pathogenesis [69], but their role is not clear. Preliminary studies reporting differences in gut microbiota composition in Graves’ patients with or without GO [70]—as well as animal studies suggesting a pivotal role of gut microbiota in TSHR‐induced disease [71]—opened an interesting field of research [72].

Cigarette smoking is an important modifiable risk factor [73] (Table 2). The incidence of clinically relevant GO is lower in European countries where tobacco consumption has decreased [7]. Smoking is associated with a lower [76] and slower [77] response to immunosuppressive treatment, and smoking withdrawal decreases the risk of GO development [78] (Table 2). Passive smoking might also be an important risk factor [79]. Mechanisms whereby smoking affects GO likely include increased generation of oxygen free radicals and hypoxia in the orbit, increased cytokine secretion, enhanced adipogenesis, and changes in the gut microbiome affecting the autoimmune process [7]. EUGOGO guidelines strongly recommend that physicians urge Graves’ patients to quit smoking [80].

**Table 2:** Modifiable risk factors for Graves’ orbitopathy (GO) and evidence supporting their role

Risk factor	Evidence	Author
Smoking	Higher prevalence of smokers in patients with GO than in patients without GO	Bartalena et al. [74]
	Greater chance of Graves’ smoker patients to develop severe forms of GO	Prummel and Wiersinga [75]
	Lower and slower response to treatments for GO in smokers	Bartalena et al. [76]
		Eckstein et al. [77]
	Decreased risk of developing GO in former smokers than in current smokers	Pfeilschifter and Ziegler [78]
Thyroid dysfunction	Amelioration of GO following restoration of euthyroidism	Prummel et al. [85]
	Higher proportion of uncontrolled hyperthyroidism in patients with moderate‐to‐severe GO than in those with mild GO	Prummel et al. [86]
	GO occurrence during a period of uncontrolled hypothyroidism	Karlsson et al. [87]
	Decreased progression of GO after RAI treatment if hypothyroidism is controlled promptly	Perros et al. [94] Tallstedt et al. [96]
RAI treatment	RAI‐associated progression of GO in 15%–20% of cases	Tallstedt et al. [90] Bartalena et al. [91]
	RAI‐associated progression of GO more likely in smokers	Bartalena et al. [91] Traisk et al. [92]
TSH‐R‐Ab	TSHR‐Ab are higher in patients with GO than in those without GO and correlate with GO activity and severity	Lytton et al. [29]
	Increased risk of GO if TSHR‐Ab are high	Khoo et al. [101]
Oxidative stress	Increased oxidative stress in patients with Graves’ disease and GO	Bartalena et al. [103]
	Increased concentrations of reactive oxygen species in blood, tears, and urine of GO patients	Hou et al. [104]
Hypercholesterolemia	High total and LDL cholesterol associated with the presence of GO	Sabini et al. [109]
	Reduced risk of GO in patients receiving statin treatment	Stein et al. [111] Nilsson et al. [112]
	Increased effectiveness of ivGCs in patients treated with atorvastatin	Lanzolla et al. [114]

Abbreviations: ivGCs, intravenous glucocorticoids; LDL, low‐density lipoprotein; RAI, radioactive iodine; TSHR‐Ab, TSH receptor antibodies.

Thyroid dysfunction is another major risk factor (Table 2). Although most GO patients have hyperthyroidism [81, 82], some of them have euthyroidism or hypothyroidism [83, 84]. In a prospective study, GO remained stable in 54 patients with euthyroidism when first seen, while it progressively improved in 33 patients with hyperthyroidism following restoration of euthyroidism [85]. Uncontrolled hyperthyroidism is more frequent in patients with moderate‐to‐severe GO than in those with mild GO [86]. Hypothyroidism is also relevant, as GO developed in 15 of 30 referred patients during a period of uncontrolled hypothyroidism [87]. In a cohort of 700 patients with chronic autoimmune thyroiditis, overt GO was present in 6% of patients, 68% of whom had circulating stimulatory TSHR‐Ab [88]. Both hyper‐ and hypothyroidism activate TSHR through TSHR‐Ab and TSH, respectively, thereby inducing an increased expression of thyroid/orbital antigens and worsening of autoimmune reactions directed against them. Therefore, EUGOGO guidelines [80] and European Thyroid Association (ETA) guidelines [89] recommend that both hyper‐ and hypothyroidism should be promptly corrected, and euthyroidism stably maintained [80].

Radioactive iodine (RAI) treatment may exacerbate GO (Table 2). Two randomized clinical trials (RCTs) showed that RAI treatment—at variance with antithyroid drug (ATD) treatment or thyroidectomy—bears a small but definite risk of GO progression [90, 91], particularly in smokers and in patients with preexisting mild GO [91]. A third RCT found that not only progression of preexisting GO, but also de novo development of GO may occur after RAI treatment [92]. This occurs in 15%–20% of cases [93] and is permanent in 5% [91]. This risk is minimal if GO is inactive [94] or longstanding (>5 years) [95]. Late correction of post‐RAI hypothyroidism likely plays a role in GO progression [94, 96]. Several studies [97, 98, 99] showed that steroid prophylaxis using low doses of prednisone can almost always prevent RAI‐associated GO progression. A starting dose of 0.3–0.5 mg prednisone/kg bodyweight, tapered and withdrawn after 3 months [91], was originally used, but lower doses (0.1–0.2 mg prednisone/kg bodyweight, tapered and withdrawn after 6 weeks) are similarly effective [98]. RAI likely causes progression of GO through the exacerbation of orbital autoimmune reactions following RAI‐associated cytolytic effect. Indeed, RAI treatment is followed by a long‐lasting increase in circulating TSHR‐Ab [100]. Based on strong evidence of its effectiveness and safety, and in agreement with current guidelines [80, 89], we believe that low‐dose oral prednisone prophylaxis should be used after RAI treatment in most patients, with the possible exception of those with longstanding and inactive GO [80, 89].

Using sensitive bioassays, stimulatory TSHR‐Ab are found in almost all patients with Graves’ disease, but circulating levels are threefold higher in GO patients, with a strong correlation with GO activity and severity [29]. In a series of 100 consecutive Graves’ patients, the odds ratio of GO was markedly increased when serum TSHR‐Ab levels were above median, and a phenotype of high TSHR‐Ab and absent thyroid peroxidase antibodies identified a group at very high risk of developing GO [101, 102]. There are no tools to reduce or block TSHR‐Ab, but ATD treatment and thyroidectomy—contrary to RAI treatment—are associated with a progressive decline in serum TSHR‐Ab levels [100].

Increased oxidative stress is found in patients with Graves’ disease and GO [103]. Reactive oxygen species stimulate orbital fibroblast proliferation, glycosaminoglycan synthesis, and upregulation of inflammatory and noninflammatory mediators [104] (Table 2). Increased concentrations of reactive oxygen species have been reported in the blood, tears, and urine of GO patients [104]. Selenium is a trace element relevant for the thyroid because selenocysteine is incorporated into selenoproteins such as iodothyronine deiodinases—involved in thyroid hormone deiodination—or thioredoxin reductase and glutathione peroxidase [105], which exert antioxidant effects [106]. In addition, selenium has immunomodulating properties [107]. In a EUGOGO multicenter RCT, selenium supplementation effectively improved mild and active GO and prevented progression to more severe forms [108], as is detailed further on.

Hypercholesterolemia is a novel risk factor (Table 2). High total and low‐density lipoprotein cholesterol levels have been associated with the presence of GO [109, 110]. Two large retrospective studies—one based on an insurance database from the United States [111] and the other on a Swedish national register [112]—showed that Graves’ patients receiving statin treatment for hypercholesterolemia had a decreased risk of GO occurrence. Finally, a single‐center, retrospective study of consecutive patients with active moderate‐to‐severe GO found that high low‐density lipoprotein cholesterol levels increased the likelihood of a poor response to intravenous glucocorticoid (ivGC) treatment [113]. More recently, a phase II, open label, single‐center RCT of 88 patients with active moderate‐to‐severe GO and increased low‐density lipoprotein cholesterol levels demonstrated that adding atorvastatin to ivGCs led to a better treatment outcome compared to ivGC monotherapy [114]. There are two relevant open questions: (i) Might statin treatment be an add‐on treatment in patients with active moderate‐to‐severe GO and normal cholesterol levels? And (ii) might statins be given to Graves’ patients with absent/mild GO, independently of cholesterol levels, to prevent GO progression [115]? Further studies are needed to address these issues. For the time being, it seems reasonable to follow the suggestion of guidelines [80] and correct hypercholesterolemia in all patients with newly diagnosed Graves’ disease, independently of the absence, presence, or degree of GO.

### Triggering events

The postpartum period is a triggering event for Graves’ hyperthyroidism [116]. Postpartum‐associated rebound immune phenomena may also favor relapse of hyperthyroidism [117, 118]. Among women in remission after ATD treatment, relapse occurred more frequently in women who then had a successful pregnancy than in those who had no pregnancy [119], although women with hyperthyroidism relapsing in the postpartum period had a greater decrease in TSHR‐Ab levels and a higher rate of remission compared to women whose relapse of hyperthyroidism was independent of pregnancy/being postpartum [120]. As far as GO is concerned, no differences in GO prevalence and degree of severity were found in women whose hyperthyroidism relapsed in the postpartum period [120]. Thus, the postpartum period is a triggering event for Graves’ disease, but not specifically for GO.

Graves’ disease and GO might be activated by infections through various mechanisms, including molecular mimicry, aberrant induction of HLA antigens, and interaction with autoantigens [121, 122]. During SARS‐CoV‐2 infection, thyrotoxicosis has been reported, mainly due to subacute/atypical (painless) thyroiditis [123, 124, 125, 126, 127, 128, 129, 130, 131]. SARS‐CoV‐2 may enter cells through the angiotensin‐converting enzyme (ACE‐2) receptor. ACE‐2 receptor mRNA has been detected both in thyroid tissue samples and in primary thyroid cell cultures [132]. In addition, the SARS‐CoV‐2 genome was reported in 36% of thyroid samples from autopsies of patients who died of COVID‐19 [133] and in one case of primary thyroid sarcoma [134]. Thus, thyrotoxicosis might be related to direct thyroid infection. Alternatively, it might result from cytokine‐induced thyroid damage [131, 132, 133, 134, 135]. Graves’ disease has been reported more rarely in patients with SARS‐CoV‐2 infection [136, 137, 138]. Three of the five patients described thus far were in long‐term remission of hyperthyroidism after ATD treatment, while two patients received a new diagnosis of Graves’ disease [136, 137, 138]. Longstanding inactive GO—previously treated with glucocorticoids (GCs)—was described in one patient, with no reactivation following SARS‐CoV‐2 infection [137]; one patient developed hyperthyroidism with mild GO [138]; and the remaining three patients presumably had no GO. Cases of thyrotoxicosis apparently induced by SARS‐CoV‐2 vaccination have also been described. In most instances, thyrotoxicosis was due to subacute/atypical thyroiditis [139]. For the time being, 25 cases of Graves’ disease have been reported after vaccination [140, 141, 142, 143, 144, 145, 146, 147, 148, 149, 150, 151, 152, 153]. Interestingly, most cases occurred shortly after the first or second vaccination using mRNA‐based vaccines, were generally mild‐to‐moderate in severity, and were easily controlled with ATDs. Reactivation of Graves’ disease after long‐term remission (7 and 17 years) following ATD treatment was reported in two cases after vaccination [149, 153]. More pertinent to this review, only one patient developed moderate‐to‐severe GO [144], while two additional patients had minimal‐to‐mild ocular involvement [149, 152]. Possible mechanisms for Graves’ disease occurrence after vaccination might include autoimmune/inflammatory syndrome induced by adjuvants [154, 155], or molecular mimicry with cross‐talk between antibodies to SARS‐CoV‐2 and thyroid—and perhaps orbital—antigens. Most of the above studies are single case reports or small series, and the occurrence of Graves’ disease might very well be only by chance. In any case, the risk of GO occurrence seems extremely low.

Graves’ disease and GO may also be triggered by thyroid damage, including subacute thyroiditis [156, 157, 158, 159], RAI treatment for toxic adenoma/multinodular goiter [160, 161], ethanol percutaneous injection for toxic adenoma [162], fine needle aspiration biopsy of thyroid nodules [163], and external radiotherapy in the head and neck region [164, 165]— although a recent, large retrospective study of patients who had received neck, craniospinal, or total body irradiation for various tumors found no cases of radiation‐induced hyperthyroidism [166]. As for RAI‐associated GO progression, the underpinning mechanism is likely the release of thyroid antigens triggering or exacerbating autoimmunity.

Graves’ disease may be triggered by immune reconstitution therapy, in particular by alemtuzumab, a monoclonal antibody that targets the CD52 antigen expressed on T cells and monocytes, thereby causing marked lymphopenia, followed by a progressive recovery of lymphocyte counts [167]. Alemtuzumab has been employed with very good results in relapsing‐remitting multiple sclerosis (RRMS), but may cause thyroid dysfunction, particularly Graves’ disease. This is not surprising, because reconstitution autoimmunity is more commonly represented by autoantibody‐mediated disorders, and Graves’ hyperthyroidism and GO are driven by TSHR‐Ab [168]. Graves’ disease was reported in 22%–33% of RRMS patients treated with alemtuzumab [169, 170, 171], and was by far the most common thyroid disorder, accounting for 63% of all cases of thyroid dysfunction in a recent systematic review and meta‐analysis of seven studies (1362 patients) [172]. In the two major original studies, GO occurred in 13 out of 136 patients—accounting for less than 10% of cases of alemtuzumab‐associated Graves’ disease—and was moderate to severe in five (4%) [169, 170]. Thus, GO in patients with alemtuzumab‐associated Graves’ disease is neither more frequent nor more severe than in the general population. However, an important feature of alemtuzumab‐associated Graves’ disease is that it often fluctuates from hyperthyroidism to hypothyroidism and vice versa, in relation to the prevailing activities of stimulatory and blocking TSHR‐Ab [170]. Instability of thyroid status is a well‐known risk factor for GO [7]. Accordingly, ETA guidelines suggest that in patients with fluctuating hyperthyroidism, consideration may be given to a definitive treatment by either RAI or thyroidectomy [173].

### Natural history

**Fig. 3:**
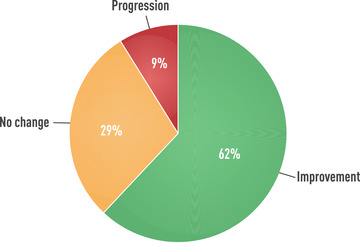
Natural history of Graves’ orbitopathy in patients with mild ocular involvement at baseline. Derived from Tanda et al. [12] and Perros et al. [175].

Mild GO involves an initial inflammatory period underpinned by ongoing orbital activation of autoimmune reactions (active phase). GO then stabilizes when inflammation starts to subside (plateau or static phase) and progressively remits following burning out of inflammation (inactive phase), possibly associated with fibrotic changes [4]. Extraocular muscle enlargement seems to occur earlier than orbital fat expansion [174]. Studies have shown that mild/minimal GO rarely progresses to more severe forms, whereas stabilization and remission are frequent [12, 13, 175, 176] (Fig. 3). In a retrospective study of 226 patients with initially active moderate‐to‐severe GO, reevaluated 4 years after GO onset and various nonsurgical and surgical treatments, further improvement was reported in 60% of responders to treatment at the last visit [177]. In a placebo group of the second teprotumumab study, at 6‐month assessment a reduction in exophthalmos ≥2 mm was observed in 10% of patients, an amelioration in diplopia in 29%, and a clinical activity score (CAS) 0–1 (inactive GO) in 21% [178]. Therefore, patients with moderate‐to‐severe GO may have the same natural history as patients with mild GO. This by no means implies that management of patients with moderate‐to‐severe GO can be delayed, because immunosuppressive treatments are largely ineffective in patients with longstanding disease.

### Early referral to specialists and thyroid eye disease clinics

Early diagnosis and management of Graves’ disease and GO is fundamental because: (i) Prompt correction of thyroid dysfunction is associated with a lower risk of progression of initially mild or absent GO [12, 85] and (ii) a favorable outcome of immunosuppressive treatment for more severe forms of GO is inversely correlated with disease duration [4, 179]. In a nationwide UK survey, correct diagnosis of GO was delayed by up to 1 year in one fourth of patients, because mild ocular inflammatory symptoms were misdiagnosed as conjunctivitis or allergic phenomena [180]. For many years, a major goal of EUGOGO has been to improve patient care through the establishment of thyroid eye disease outpatient clinics run by endocrinologists and ophthalmologists/orbital surgeons, with interdisciplinary collaboration of other specialists such as orthoptists, thyroid surgeons, radiotherapists, and radiologists [181]. Satisfaction regarding the management of GO is higher among patients who attend such specialized centers [180]. Propagation of this interdisciplinary approach introduced by EUGOGO contributed to a decrease in patient referral time and reduced GO severity of patients referred to EUGOGO tertiary centers over a 12‐year period [14]. Helpful indications on the management of GO outside specialized centers and referral pathways have been published [182]. According to EUGOGO guidelines, primary‐care physicians, general practitioners, general internists, and specialists should promptly refer all patients with overt GO or at risk of progression (e.g., smokers and those with unstable hyperthyroidism or high TSHR‐Ab levels) to thyroid eye clinics or specialized centers [80].

## Assessment

The main features of GO to be assessed are activity and severity because both contribute to identifying and prescribing appropriate treatments according to the phase and degree of orbital involvement.

**Fig. 4:**
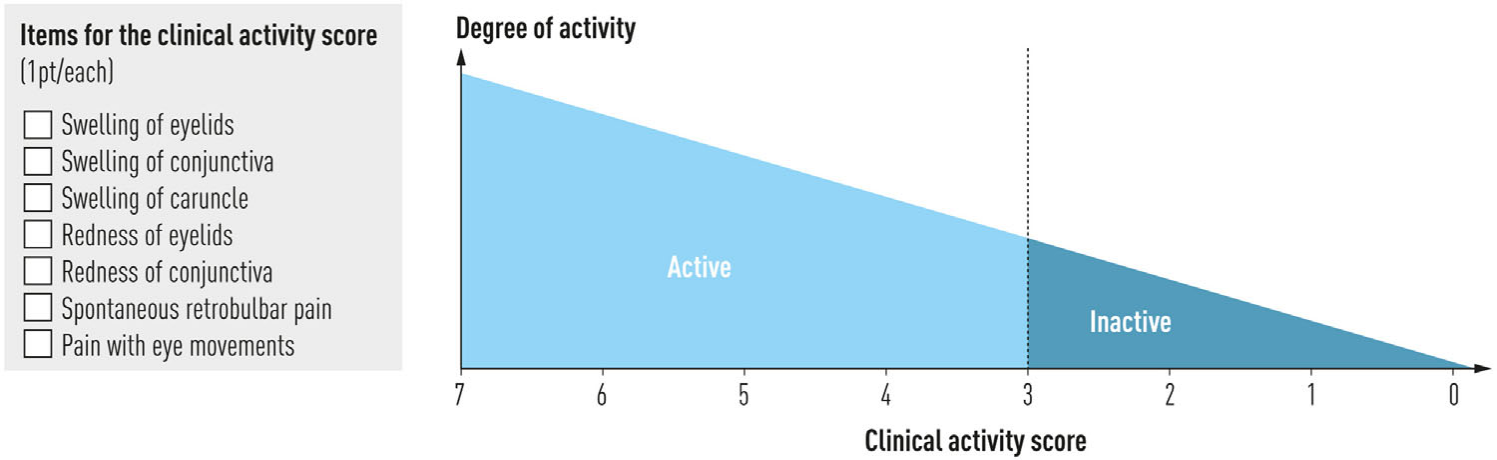
Assessment of activity of Graves’ orbitopathy by the clinical activity score.

CAS—a scoring system that substantially evaluates inflammation [183]—is the best validated and most used tool to assess GO activity. CAS includes seven items, with one point given to each item present. GO is defined as active if the sum is ≥3/7 (Fig. 4). A 10‐item version of the CAS includes three additional features—increase in exophthalmos of ≥2 mm, decrease in eye motility, and decrease in visual acuity—that are useful in defining recent (1–3 months) GO progression. CAS is not perfectly objective because two items—spontaneous and gaze‐evoked pain—are subjective. In addition, CAS is binary—indicating presence or absence, without grading—but an image atlas may help to improve the consistency of CAS assessment [184]. Thus, CAS is a useful and quick tool, easily applicable in daily clinical practice to assess the activity of GO and to monitor the effects of treatment on inflammation. Other scores, such as the VISA (Vision, Inflammation, Strabismus, Appearance/exposure) score [185], have been proposed, but they need more thorough validation. Magnetic resonance imaging may also be useful, but it is expensive and not always available.

Measures of severity include assessment of lid aperture by a ruler, exophthalmos by a Hertel exophthalmometer, ocular motility by ductions, corneal status by slit lamp [181], and assessment of optic nerve status by visual acuity, visual fields, color vision, afferent pupil defects, fundus oculi evaluation, and optical coherence tomography [186]. A precise definition of GO severity is somehow elusive, but EUGOGO has proposed a classification into mild, moderate‐to‐severe, and sight‐threatening—very severe—GO, based on the combination of different features [80] (Table 3).

**Table 3:** Classification of severity of Graves’ orbitopathy

Degree of severity	Features
Mild	One or more of:
	Lid retraction <2 mm
	Mild soft tissue involvement
	Exophthalmos <3 mm above normal
	No/intermittent diplopia
Moderate to severe	Two or more of:
	Lid retraction ≥2 mm
	Moderate‐to‐severe soft tissue involvement
	Exophthalmos ≥3 mm above normal
	Inconstant/constant diplopia
Sight threatening	Presence of DON and/or corneal breakdown

*Note*: Adapted from Bartalena et al. [80].

Abbreviation: DON, dysthyroid optic neuropathy.

Standardization of assessment should be implemented to allow homogeneous reporting of treatment outcomes and, thereby, the comparison of effects of different therapies for GO [187].

### Management of mild GO

General measures include control of modifiable risk factors for GO progression—for example, refraining from smoking, tight control of thyroid dysfunction, steroid prophylaxis after RAI treatment, and correction of hypercholesterolemia [80]. Artificial tears during the day and ocular gels/ointments at night are useful to reduce symptoms due to inflammation or dry eye [188].

In an RCT of 159 patients with active mild GO, selenium administration (selenium selenite 100 µg twice a day, equivalent to 91.2 µg of selenium) for 6 months induced an improvement in quality of life and overall ocular involvement significantly better than placebo [108]. Furthermore, selenium treatment effectively prevented progression of GO to more severe forms 6 months after intervention [108]. No cases of selenium toxicity were observed. Most patients came from marginally selenium‐deficient European areas; accordingly, it is not clear whether selenium supplementation is also helpful in patients living in selenium‐sufficient regions. An Italian questionnaire‐based survey revealed a wide use of selenium supplementation for conditions lacking evidence of benefit, including autoimmune thyroiditis and Graves’ hyperthyroidism without GO [189]. This misuse should be avoided [107, 190, 191]. Likewise, there is no evidence that selenium may be considered an adjuvant therapy for active moderate‐to‐severe GO treated with immunosuppressive/targeted therapies. Accordingly, current guidelines recommend a 6‐month course of selenium only for active mild GO [80]. If selenium selenite is unavailable, it can be substituted with seleniomethionine (100 µg daily).

In patients whose quality of life is markedly impaired despite mild features of active GO, a course of low‐dose ivGCs can be considered within a shared decision‐making process [80]. Likewise, in patients with residual manifestations of mild and inactive GO (lid malposition, minimal exophthalmos), rehabilitative surgery can also be considered, as needed or requested by the informed patient [80].

### Management of active moderate‐to‐severe GO

Active moderate‐to‐severe forms of GO affect a minority of patients but are a major challenge and dilemma because established medical treatments often achieve incomplete results [192, 193]. GCs still constitute the mainstay in the management of active moderate‐to‐severe and sight‐threatening GO [80], but recent advances using either old drugs with new applications or novel biological agents have expanded the armamentarium available for treatment [194, 195].

### GCs as monotherapy or in combination with other therapies

GCs have been used for decades for their potent anti‐inflammatory and immunosuppressive effects achieved through interference with T‐ and B‐cell functions, decreased recruitment of monocytes and macrophages, reduced secretion of proinflammatory cytokines and increased secretion of anti‐inflammatory cytokines, and decreased synthesis of glycosaminoglycans [195, 196]. High doses of GCs are required, and both the oral route and intravenous (iv) route can be used, while locally administered GCs are poorly effective [1]. RCTs have demonstrated that the iv route is more effective and better tolerated than the oral route [197, 198]. A proof‐of‐principle demonstration of ivGC efficacy was provided by an RCT showing favorable responses in 83% of patients treated with ivGCs versus 11% of placebo‐treated patients [199]. The small‐sized study was stopped before the end of recruitment because the difference between treatment and placebo groups was so striking that it was deemed unethical to leave patients untreated [199]. Higher efficacy of the iv route was confirmed by a systematic review and meta‐analysis [200] and an overall response rate of about 80% was reported in another systematic review [201]. In a large RCT from EUGOGO centers, the overall response rate was lower than that above [202], possibly in relation to the relatively long duration of GO and not very severe clinical features of enrolled patients. A careful selection of patients is needed, because the activity of GO—indicated by CAS—is predictive of a good response to immunosuppressive treatment, whereas a duration of GO longer than 16 months is associated with a poor response [203]. Contraindications to high‐dose systemic GC treatment include recent viral hepatitis, psychiatric disorders, and severe cardiovascular morbidity, while hypertension and diabetes are not absolute contraindications, but should be kept under control before treatment [80]. Oral GC therapy is typically instituted using 50–100 mg/day prednisone and the dose is gradually tapered and withdrawn after 6 months [4]. Most commonly, ivGCs are given as 12 weekly infusions of methylprednisolone (six using 500 mg and six using 250 mg, cumulative dose: 4.5 g) [198]. Higher doses can be used for more severe forms within the spectrum of moderate‐to‐severe GO, but the cumulative dose should not exceed 8 g per cycle, and a single dose should not be >750 mg—with possible exception of DON—to minimize risks of major adverse events [204]. Infusion should be slow (1–2 h) under close surveillance [80]. If facilities are not available, oral GCs represent an acceptable alternative [205]. Response to treatment usually occurs early but may become evident in the second half of the course [206, 207]. Inflammatory signs and symptoms are highly responsive, improving in 59%–83% of cases in a large RCT [202]. In a systematic review of seven RCTs, ocular motility improved in 57% of cases and diplopia improved/disappeared in 64% [201]. Exophthalmos is the least responsive feature, with a mean decrease of 1.14 mm in RCTs and 1.58 mm in non‐RCTs [201]. A limitation of GC treatment is relapse after drug discontinuation, occurring in about 10% of cases [194]. Adverse events are relatively common [208], although usually minor to moderate [201] in severity. The most threatening adverse event is severe hepatotoxicity, more frequent in the past when much higher single dose and cumulative doses (10–24 g) of methylprednisolone were used [209, 210]. In a comprehensive review published more than 10 years ago, overall morbidity was 6.5% and mortality 0.6% (six patients out of 1045, all treated with doses of GCs higher than currently used) [201].

Mycophenolate inhibits proliferation of both T and B cells and antibody and adhesion molecule formation, thereby modulating tissue inflammation. For these actions, mycophenolate mofetil is used to prevent transplant rejection [211] and for autoimmune disorders. Most relevant adverse events of mycophenolate mofetil are gastrointestinal, but enteric‐coated tablets of mycophenolate sodium are better tolerated [212]. One gram of mycophenolate mofetil is equivalent to 720 mg of mycophenolate sodium [212]. Mycophenolate mofetil was used for active moderate‐to‐severe GO in a single‐center RCT from China in which 80 patients were treated with mycophenolate mofetil monotherapy (500 mg twice a day for 24 weeks) and 78 patients with high‐dose GC monotherapy (an unusual regimen of 500 mg iv methylprednisolone for three consecutive days for 2 weeks, followed by oral prednisone, 60 mg starting dose, gradually tapered down and withdrawn at week 24) [213]. Both treatments were effective, but mycophenolate mofetil provided better results than GC monotherapy in terms of improvement of CAS, diplopia, and exophthalmos (mean reduction at 24 weeks: 3.2–3.4 mm vs. 1.9–2.2 mm after GCs). In a retrospective study of 20 patients with moderate‐to‐severe to sight‐threatening GO, mycophenolate mofetil was successfully used as a second‐line treatment in patients previously treated with ivGCs [214]. In a subsequent multicenter European RCT, 81 patients were allocated to receive ivGC monotherapy (cumulative dose: 4.5 g) and 83 patients to receive combined therapy with the same ivGC dose plus mycophenolate sodium (720 mg daily orally for 24 weeks) [215]. Although variation of a composite index of ocular assessment at 12 weeks (end of GC treatment)—indicated as primary outcome—improved significantly in both groups, with no significant difference between groups, analysis at 24 weeks (end of mycophenolate sodium treatment) and 36 weeks (3‐month follow‐up) clearly showed that combination therapy was more effective in terms of overall response and changes of individual features [215]. At variance with the Chinese study, response of exophthalmos was less impressive. Treatment was generally well tolerated, and neither dose reduction nor drug withdrawal because of drug‐related side effects was needed.

Orbital radiotherapy (OR) is a well‐established treatment for GO due to anti‐inflammatory effects and radiosensitivity of lymphocytes infiltrating the orbit [216]. In a double‐blind RCT of 60 patients with active moderate‐to‐severe GO, 30 patients were treated with OR (20 Gy subdivided into 10 doses of 2 Gy) and 29 were sham irradiated [217]. Favorable responses were achieved in 60% of irradiated patients and in only 31% of sham‐irradiated patients [217]. The difference between groups was particularly evident for ocular motility and related diplopia [217]. Another RCT of patients with mild GO confirmed OR effectiveness, particularly in patients whose disease duration was ≤18 months, and, again, especially in the presence of ocular dysmotility and diplopia [218]. A third RCT provided negative results, but this study was limited by a long duration of GO and previous unresponsiveness to GCs, which introduced a serious selection bias [219]. OR effectiveness was also demonstrated in an RCT of 56 patients treated either with oral GCs and sham irradiation or with OR and placebo [220]. Two RCTs investigating a combination of OR and oral GCs versus either oral GCs or OR monotherapy [179, 221] showed that combined treatment was more effective than either treatment alone. A study from the UK reported that adding OR to oral prednisolone did not provide substantial benefits [222], but a recent systematic review and meta‐analysis of published literature showed that OR is effective in selected patients, particularly those with disturbances of ocular motility and in combination with GCs [223]. Combination of OR with ivGCs is more effective than combination of OR with oral GCs [197]. In addition, two retrospective studies have shown that ivGCs combined with OR are more effective than ivGC monotherapy, particularly in improving ocular motility and reducing GO severity [224, 225]. The most common schedule of OR is 10 daily doses of 2 Gy over a 2‐week period, but a weekly dose of 1 Gy for 20 weeks may be equally effective [226]. OR is safe [227, 228] but it should be avoided in patients with hypertensive or diabetic retinopathy because of related microvascular lesions, and it is usually not used in patients younger than 35 years for a remote carcinogenic potential [216].

Cyclosporine is a potent immunosuppressive drug acting on both humoral and cellular immunity. In a single‐center RCT of 40 patients with active moderate‐to‐severe GO, cyclosporine (starting dose: 5–7.5 mg/kg body weight; duration of treatment: 6 months) used in combination with oral prednisone was more effective than prednisone monotherapy and associated with a lower relapse rate [229]. Several cases of a reversible rise in blood pressure and liver enzymes and one case of *Klebsiella pneumoniae* pneumonia were observed [229]. A second, single‐blind single‐center RCT of 36 patients compared oral prednisone monotherapy and cyclosporine monotherapy (dose: 7.5 mg/kg body weight) over a period of 3 months [230]. Responders were 61% among prednisone‐treated patients and only 22% among cyclosporine‐treated patients [230]. However, when nonresponders of either group were given a second treatment with a combination of the two drugs, around 60% of initial nonresponders significantly improved [230]. Cyclosporine was better tolerated than prednisone, with six cases of reversible hypertension and one case of irreversible rise in the plasma creatinine concentration [230]. No RCTs have investigated the combination of ivGCs and cyclosporine.

Azathioprine is used for GO but its role is uncertain. In an old prospective study (10 patients treated with azathioprine and 10 untreated), azathioprine had no beneficial effects on several ophthalmic parameters [231]. Conversely, in a recent RCT, the addition of azathioprine to oral prednisolone was in post‐hoc analysis associated with an improved outcome compared to oral prednisolone alone, but results were affected by the large number of patients who withdrew from the study because of drug‐related adverse events [222].

Methotrexate has been reported for the management of GO in small and retrospective studies [232, 233, 234], and no sound conclusions can be drawn on its role.

Combination of GCs with other immunosuppressive drugs or therapies is interesting because combined therapy may potentiate the effects of GC monotherapy and have a steroid‐sparing effect [234, 235, 236].

### Biological agents

Targeted therapies aimed at intervening on pivotal steps of GO pathogenesis have recently been investigated [194] with promising results, triggering a debate on whether they may replace GCs as first‐line treatment for active moderate‐to‐severe GO [237] (Table 4).

**Table 4:** Biological agents investigated in randomized trials for the treatment of active moderate‐to‐severe Graves’ orbitopathy

Agent	Target	Effectiveness	Safety	Durability	Cost
Teprotumumab	IGF‐1 receptor	Yes	Long‐term safety unknown	Frequent relapses	Very high
Rituximab	CD20‐positive B cells	Conflicting^a^	Long‐term safety unknown	Yes	High
Tocilizumab	IL‐6 receptor	Yes^b^	Long‐term safety unknown	Unknown	High

Abbreviations: IGF‐1, insulin‐like growth factor‐1; IL‐6, interleukin‐6.

^a^ Two small randomized clinical trials, one with negative results (vs. placebo) and one with positive results (same effectiveness as intravenous glucocorticoids, better durability).

^b^ In glucocorticoid‐resistant Graves’ orbitopathy.

Teprotumumab is a fully humanized monoclonal antibody blocking IGF‐1R. Blocking IGF‐1R and its cross‐talk with TSHR may be relevant to arresting GO development/progression [36]. In a first double‐masked, multicenter RCT, 87 patients were enrolled, of whom 76 (39 in the placebo group, 37 in the teprotumumab group) completed the 24‐week intervention [238]. Teprotumumab treatment consisted of eight iv infusions at 3‐week intervals (starting dose: 10 mg/kg bodyweight, then increased to 20 mg/kg bodyweight); the primary composite end point (response) included a CAS reduction ≥2 points and decrease in exophthalmos ≥2 mm [238]. At 24 weeks, 69% of teprotumumab‐treated patients and 20% of placebo‐treated patients had a positive response [238]. The most striking effect was on exophthalmos, with a mean reduction of about 2.5 mm (compared to 0.15 mm in the placebo group), and 40% of patients showing a decrease of ≥4 mm [238]. Positive results in the teprotumumab group included CAS reduction averaging 3.4 points, with 69% of patients showing a CAS of 0–1 point at week 24 (vs. 21% in the placebo group) [238]. The most severe grades of diplopia (inconstant and constant) were present at baseline in 52% of patients but persisted at week 24 in 31% of patients. Likewise, improvement in the quality of life occurred, less impressively, in both visual functioning and appearance subscales [238]. A subsequent double‐masked, placebo‐controlled phase 3 multicenter RCT from the same investigators (OPTIC trial, 41 patients treated with teprotumumab, 42 with placebo) had as the primary outcome a decrease in exophthalmos ≥2 mm [178]. Response to teprotumumab was observed in 83% versus 10% of placebo‐treated patients and secondary outcomes (CAS, diplopia score, quality of life) were significantly better in the teprotumumab group [178]. These results in patients with active moderate‐to‐severe GO convinced the US Food and Drug Administration to approve teprotumumab in January 2020 for GO treatment, irrespective of grade (mild, moderate, sight threatening) or state (active, inactive). To assess the durability of therapeutic response, the same investigators performed a pooled data analysis of the two studies and an off‐treatment follow‐up [239]. At a longer follow‐up (72 weeks), exophthalmos relapsed in 33% of patients, diplopia in 31%, and using a composite overall evaluation, 17% could no longer be considered responders [239]. In a recent study (OPTIC‐X), OPTIC nonresponders or those who flared (37 in placebo group and 14 in the teprotumumab group) were treated or retreated with teprotumumab, and a response was achieved in the majority of cases [240]. It should be noted that the cost of a teprotumumab course is exceedingly high, in the order of a few hundred thousand US dollars, compared to a few hundred US dollars for ivGCs. In addition, teprotumumab has not been approved by the European Medicines Agency. Another relevant issue, as with any novel drug, is safety. In the two registration studies, teprotumumab was relatively safe, although several adverse events, usually mild to moderate—including muscle spasms, diarrhea, and hyperglycemia—were reported [178, 238]. In the post‐marketing period, one case of amyloid encephalopathy [241] and two cases of new inflammatory bowel disease (ulcerative colitis) [242, 243] have been described. Even more worrisome is the report of permanent sensorineural hearing loss, so far described in 13 of 190 patients from five series [244]. This warrants a careful identification of risk factors and careful on‐treatment and post‐treatment monitoring of patients treated with teprotumumab. In addition, for the time being it seems advisable to limit the use of teprotumumab to active moderate‐to‐severe GO, where it is evidence‐based, and the risk–benefit ratio is reasonable [244]. Finally, head‐to‐head comparison of ivGCs and teprotumumab in a large multicenter RCT in the same clinical setting is warranted and cannot be bypassed.

Rituximab is a chimeric mouse–human monoclonal antibody targeting CD20‐positive B cells; its immunosuppressive action is related to B‐cell depletion [245], although it also causes a decrease of IGF‐1R^+^ T cells [246]. Rituximab was initially reported in small and uncontrolled studies to be beneficial for active moderate‐to‐severe GO, especially when resistant to GC treatment [247, 248, 249]. Then, two small single‐center RCTs were published—from the Mayo Clinic in the United States and from Italy [249, 250]. In the Mayo Clinic RCT, 25 patients were randomized to receive either rituximab (two 1000‐mg infusions at a 2‐week interval) or saline. The primary outcome was CAS change at 24 weeks, and secondary outcomes were changes in several ocular parameters and quality of life. Eleven patients in the rituximab group and 10 in the placebo group completed the study [250]. This study failed to find significant differences in treatment outcomes between groups [250]. In the Italian RCT, 32 patients were randomized to receive either ivGCs (cumulative dose, 7.5 g) or rituximab (two 1000‐mg infusions at a 2‐week interval for the first 12 patients, then a single 500‐mg infusion for the last four patients). The primary outcome was a decrease in CAS ≥2 points or disease inactivation (final CAS <3) [251]. At 24 weeks, 100% of rituximab‐treated patients versus 69% of GC‐treated patients improved, and no patient in the rituximab group had subsequent GO reactivation, observed in one third of GC‐treated patients [251]. An attempt was made to reconcile these conflicting results, which might somehow be explained by longer GO duration in the Mayo Clinic study, making patients less responsive [252]. In the above studies, DON occurred in two patients and vasculitis in one patient treated with rituximab [250], and severe cytokine release syndrome (severe periorbital swelling and decreased vision)—reverted by ivGCs—in two patients [251]. A retrospective multicenter nationwide French study reported an efficacy of rituximab as intermediate between the two RCTs [253]. A meta‐analysis of 12 studies (152 patients) concluded that rituximab has a beneficial effect on CAS (inflammation) and limited efficacy on exophthalmos [254]. Recently, a small and uncontrolled study of 17 GC‐resistant patients reported that, after a single 100‐mg rituximab infusion, GO inactivation occurred in more than 90% of patients, and severity improved in about 60%, with no flares [255]. To summarize, evidence from the small RCTs is conflicting, and larger multicenter RCTs are needed. For the time being, rituximab may represent a valid option as a second‐line treatment for active moderate‐to‐severe GO.

Tocilizumab is a humanized monoclonal antibody targeting interleukin‐6 (IL‐6) receptor, approved for treatment of rheumatoid arthritis. IL‐6 is a proinflammatory cytokine overexpressed in orbital fibroblasts [256, 257] and capable, among other actions, of stimulating TSHR expression in human orbital preadipocyte GO fibroblasts [258]. Following a few small uncontrolled studies [259, 260, 261], in a small placebo‐controlled RCT, 32 GC‐resistant GO patients were given either iv tocilizumab (8 mg/kg bodyweight, four doses at a 4‐week interval) or placebo [262]. Tocilizumab‐treated patients had a significantly higher rate of GO inactivation, but a surprisingly high rate of inactivation was also observed in placebo‐treated patients [262], suggesting that improvement may partially reflect a late effect of previous GC treatment or GO natural history. Tocilizumab had limited efficacy on exophthalmos and diplopia [262]; it was well tolerated, but there was a higher rate of infections and headache [262]. Other small uncontrolled or retrospective studies seem to confirm tocilizumab efficacy, particularly in GC‐resistant GO [263, 264, 265], but larger multicenter RCTs are warranted to better define its role. For the time being, tocilizumab may be considered as a second‐line treatment for active moderate‐to‐severe GO, particularly if refractory to ivGCs.

### Approach to the management of active moderate‐to‐severe GO

**Fig. 5:**
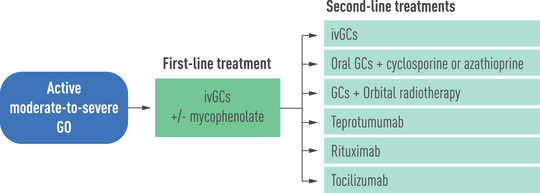
First‐line and second‐line treatments for active moderate‐to‐severe Graves’ orbitopathy, according to the European Group on Graves’ Orbitopathy clinical practice guidelines [80]. GCs, glucocorticoids; ivGCs, intravenous glucocorticoids.

High‐dose GCs, preferably given intravenously, currently represent the mainstay of treatment for active moderate‐to‐severe GO. Recently published EUGOGO guidelines recommend using iv methylprednisolone (cumulative dose: 4.5 g) in combination with mycophenolate sodium (720 mg daily for 24 weeks), while for most severe forms of GO (with inconstant/constant diplopia) within the spectrum of moderate‐to‐severe GO, a higher dose of ivGC monotherapy (cumulative dose: 7.5 g) is recommended, because evidence on using the highest dose of methylprednisolone in combination with mycophenolate is lacking [80] (Fig. 5). For the time being, the guidelines do not consider teprotumumab as an alternative first‐line treatment—despite its efficacy—because of the current lack of data on its long‐term efficacy and safety, its exceedingly high cost, and its unavailability outside the United States [80].

If response to first‐line treatment is partial or absent, second‐line treatments include: (i) a second course of ivGCs, using the highest cumulative dose (7.5 g), (ii) oral GCs combined with either cyclosporine or azathioprine, (iii) OR combined with either oral or iv GCs, (iv) teprotumumab, (v) rituximab, and (vi) tocilizumab [80] (Fig. 5). Clearly, due to rapid progress in this field, these recommendations might need to be revised in a few years.

When GO has been inactive at least 6–12 months, rehabilitative surgeries—such as orbital decompression, squint surgery, and eyelid surgery—can be performed, if needed or required by the patient, following the above order. Patient choice, within a thorough shared decision‐making process, is of the utmost importance at this stage as well as in the choice of first‐ and second‐line medical treatments.

### Management of sight‐threatening GO

Sight‐threatening GO, due to corneal breakdown or DON, is an emergency condition warranting immediate treatment. The corneal breakdown may be related to severe corneal exposure in the case of severe exophthalmos and/or lagophthalmos (incomplete eyelid closure at night). This condition requires intensive local care, using gluing, antibiotics, blepharorrhaphy, tarsorrhaphy, and lid lengthening.

In a small RCT of DON, 15 patients were randomized to immediate orbital decompression or ivGC treatment [266]. Five of nine patients treated medically did not need subsequent decompression, whereas all six patients submitted to an initial surgical treatment then required immunosuppressive treatment [266]. There was no better outcome in patients treated with immediate surgery [266]. In a retrospective study, 40% of patients initially treated with ivGCs experienced a permanent normalization of visual acuity [267]. Accordingly, current guidelines recommend the following treatment schedule: (i) administer iv methylprednisolone 500–1000 mg for three consecutive days or on alternate days, (ii) repeat the cycle the next week, (iii) in the case of a good response, continue with weekly infusion as for active moderate‐to‐severe GO, and (iv) in the case of an absent/poor response, submit the patient to urgent orbital decompression [80].

### Treatment of hyperthyroidism in patients with GO

Graves’ hyperthyroidism can be treated by ATDs, RAI, or thyroidectomy [268]. Neither ATDs nor thyroidectomy directly modify GO natural history [90, 91, 269]. Long‐term ATD treatment‐associated gradual decrease in serum TSHR‐Ab levels may be beneficial for GO. A small RCT [270] and a retrospective case study [271] reported that early thyroidectomy may result in a more favorable outcome of immunosuppressive therapy. As discussed, RAI bears a small but definite risk for GO [90, 91, 97], preventable by steroid prophylaxis [80]. Total thyroid ablation (thyroidectomy followed by RAI treatment) may be beneficial in the short run, but not in the long [272, 273, 274].

Given these premises, the optimal treatment for hyperthyroidism in patients with GO remains a dilemma. If GO—either mild or moderate to severe—is stable and longstanding as inactive, any treatment for hyperthyroidism can be chosen, as it is unlikely to cause GO recurrence/progression [80]. In patients with residual but longstanding inactive moderate‐to‐severe GO, steroid prophylaxis can be considered if RAI treatment is selected and risk factors are still present [80]. If GO is active and mild, ATD treatment (or thyroidectomy) is preferred, but RAI treatment can also be selected, with steroid prophylaxis [80]. In the presence of active moderate‐to‐severe GO, treatment of GO should be prioritized, as delayed treatment is associated with a worse response to immunosuppressive treatment [80]. While GO is being treated, hyperthyroidism is controlled by ATD treatment, which can be continued for several years until GO is cured [275, 276]. If GO is sight threatening, its emergency treatment—either medical and/or surgical—is an absolute priority. Hyperthyroidism is stabilized by ATDs until treatment of GO is completed [80].

## Conclusions and perspectives

Recent years have witnessed the development of novel therapies targeting different crucial steps/sites for GO development: B cells as antigen‐presenting cells and autoantibody producers (rituximab), IGF‐1R (teprotumumab), and IL‐6 receptor (tocilizumab). The results are certainly promising, for some drugs more than for others, but they need to be corroborated by larger studies. And, given the complexity of the autoimmune orbital process, other biological agents—such as tumor necrosis factor α inhibitors (etanercept, infliximab, adalimumab, belimumab)—interfering with a cytokine cascade might prove to be effective.

Iscalimab—a monoclonal antibody targeting CD40 (found on the surface of antigen‐presenting cells and thyrocytes), thus interrupting CD40 interaction with its ligand (CD154) and, thereby, abating autoimmune response—was used in a phase II study of 15 Graves’ patients with normalization of thyroid status and decrease in serum TSHR‐Ab levels in 47% [277]. In view of its mechanism of action, this agent might in the future prove beneficial for GO as well.

As loss of tolerance to TSHR is central in the pathogenesis of both Graves’ hyperthyroidism and GO, antigen‐specific immunotherapy is a fascinating goal. Retolerization by repeated injections of small and increasing amounts of synthetic peptides based on a sequence of TSHR (ATX‐GD‐59) restored euthyroidism in five of 10 patients in a phase I study [278]. If these preliminary results are confirmed in larger studies, it is conceivable that this approach might also be useful for GO.

Small molecule/peptides binding to transmembrane portions of TSHR and, thereby, antagonizing signaling induced by TSHR‐Ab may represent a future treatment for both hyperthyroidism and GO, but studies are thus far preclinical [279, 280, 281].

A blocking TSHR‐Ab, K1‐70, effectively blocked a stimulating monoclonal TSHR‐Ab, M22, in an animal model [282]. K1‐70 was administered to a 51‐year‐old woman affected by Graves’ disease associated with high stimulatory TSHR‐Ab levels, GO, and metastatic differentiated thyroid cancer [283]. Following K1‐70 administration, serum‐blocking TSHR activity increased, and this was associated with an improvement of GO and stabilization of metastatic lesions (during an interval of lenvatinib therapy) [283]. More recently, in an open‐label phase I study, 18 Graves’ patients—euthyroid or hyperthyroid under ATD treatment—were treated with a single shot of either intramuscular or iv K1‐70 [284]. Patients were allocated to six cohorts of three patients each and given increasing doses of K1‐70 (0.25–150 mg) and followed for 100 days [284]. On day 28, most patients—especially in the high‐dose cohorts—progressed to a hypothyroid state, with improvement of symptoms that persisted in many instances at the end of follow‐up [284]. Interestingly, two patients with associated GO experienced improvement of GO symptoms and signs, including a marked reduction in exophthalmometer readings [284]. These results, although preliminary, suggest that K1‐70 might represent a TSHR‐specific drug with a potential for managing both Graves’ hyperthyroidism and GO.

## Conflict of interest

The authors declare there is no conflict of interest that could be perceived as prejudicing the impartiality of the research reported. This paper was not funded.
